# Evaluating the reproducibility of a deep learning algorithm for the prediction of retinal age

**DOI:** 10.1007/s11357-024-01445-0

**Published:** 2024-11-26

**Authors:** Jay Rodney Toby Zoellin, Ferhat Turgut, Ruiye Chen, Amr Saad, Samuel D. Giesser, Chiara Sommer, Viviane Guignard, Jonas Ihle, Marie-Louise Mono, Matthias D. Becker, Zhuoting Zhu, Gábor Márk Somfai

**Affiliations:** 1 Department of Ophthalmology, Stadtspital Triemli: Stadtspital Zurich Triemli, Birmensdorferstrasse 497, CH-8063 Zurich, Switzerland; 2 Spross Research Institute, Zurich, Switzerland; 3 Centre for Eye Research Australia, Royal Victorian Eye and Ear Hospital, Melbourne, Australia; 4 Department of Neurology, Stadtspital Triemli: Stadtspital Zurich Triemli, Zurich, Switzerland; 5 Department of Ophthalmology, University of Heidelberg, Heidelberg, Germany; 6 Department of Ophthalmology, Semmelweis University, Budapest, Hungary; 7 Gutblick Research, Pfäffikon, Switzerland

**Keywords:** Retinal age gap, Deep learning algorithm, Retinal imaging

## Abstract

Recently, a deep learning algorithm (DLA) has been developed to predict the chronological age from retinal images. The Retinal Age Gap (RAG), a deviation between predicted age from retinal images (Retinal Age, RA) and chronological age, correlates with mortality and age-related diseases. This study evaluated the reliability and accuracy of RA predictions and analyzed various factors that may influence them. We analyzed two groups of participants: Intravisit and Intervisit, both imaged by color fundus photography. RA was predicted using an established algorithm. The Intervisit group comprised 26 subjects, imaged in two sessions. The Intravisit group had 41 subjects, of whom each eye was photographed twice in one session. The mean absolute test–retest difference in predicted RA was 2.39 years for Intervisit and 2.13 years for Intravisit, with the latter showing higher prediction variability. The chronological age was predicted accurately from fundus photographs. Subsetting image pairs based on differential image quality reduced test–retest discrepancies by up to 50%, but mean image quality was not correlated with retest outcomes. Marked diurnal oscillations in RA predictions were observed, with a significant overestimation in the afternoon compared to the morning in the Intravisit cohort. The order of image acquisition across imaging sessions did not influence RA prediction and subjective age perception did not predict RAG. Inter-eye consistency exceeded 3 years. Our study is the first to explore the reliability of RA predictions. Consistent image quality enhances retest outcomes. The observed diurnal variations in RA predictions highlight the need for standardized imaging protocols, but RAG could soon be a reliable metric in clinical investigations.

**Supplementary Information:**

The online version contains supplementary material available at 10.1007/s11357-024-01445-0.

## Introduction

Aging drives molecular and physiological changes in the human body, substantially increasing the risk of multiple age-related diseases and all-cause mortality [1–4]. However, due to significant interindividual variability in aging rates, the correlation between chronological age and the underlying biological aging processes influencing disease susceptibility is weak, limiting its utility as a standalone predictive biomarker [5–8]. Hence, the concept of biological age has emerged to better capture cumulative molecular damage, cellular changes, and decline in physiological reserves [9–11]. Several biomarkers of biological age have been proposed, including epigenetic clocks, transcriptomic predictors, and imaging-based markers [12, 13]. Still, many of the emerging biomarkers of biological age have significant limitations, including invasiveness, high cost, intensive testing requirements, and low availability [11, 12]. This has driven the development of novel noninvasive biological age biomarkers.

To this end, recent advances in deep learning (DL) have enabled the design of several deep learning algorithms (DLA) trained to predict age from fundus images, called Retinal Age (RA) [14–17]. Studies have found that an increased positive deviation of predicted RA from chronological age, defined as the Retinal Age Gap (RAG), is significantly associated with increased all-cause mortality, and age-related diseases, such as cardiovascular events [14–25]. Accordingly, DLA-predicted RA may serve as a valuable, easily assessable, and highly relevant outcome in a wide array of clinical trials in the near future. However, while promising, there is still no evidence available on the reproducibility and retest reliability of these predictions. Thus, it is still unknown whether RA predictions are consistent or exhibit substantial variations within short periods, making power analysis for clinical trials difficult, and hindering effective sample-size calculations. Additionally, no strict guidelines for the acquisition of fundus images used for RA prediction in clinical trials have been defined, and there is a complete lack of research exploring different image quality and patient-related factors that may affect their precision.

For the above reasons, the primary aim of this study was to determine the retest reliability (precision) and reproducibility of RA predictions by the DLA most extensively validated for its association with clinical outcomes. Our secondary objective was to investigate the accuracy of the DLA in predicting chronological age from a collection of fundus images from healthy individuals not included in the training and validation dataset. Additionally, we aimed to investigate the effect of different variables on the precision of RA predictions, including circadian changes and image-related metrics. We also explored inter-eye variance in RA predictions and investigated, whether the deviation of perceived subjective age from chronologic age is correlated with RAG. Finally, we utilized the open-source deep learning tool, AutoMorph [26], to extract features of image quality and vascular morphology from retinal fundus images, allowing investigation of potential interactions between these features and the retest reliability of the RA predictions.

## Methods

### Study design and population

We conducted a non-randomized study with two groups to determine the retest reliability, reproducibility, precision, and accuracy of RA measurements. The first group consisted of 26 healthy individuals, 45–65 years of age, recruited from the outpatient clinics at the Department of Ophthalmology of Stadtspital Zürich, Zurich, Switzerland, to assess the Intervisit reproducibility of RA measurements (Intervisit Group). Only patients with a negative ocular history and up to one cardiovascular risk factor (CVR) were included (BMI > 30 kg/m^2^, consistently elevated blood pressure > 130/80 mmHg or arterial hypertension > 140/90 mmHg, diabetes mellitus type 2, persistent nicotine use). The second group included 41 healthy adult volunteers, 22–59 years of age, and with a negative history of diabetes and rheumatologic diseases. These participants were recruited anonymously from the employees and visitors of Stadtspital Zürich, Zurich, Switzerland to evaluate the retest reliability of RA measurements in a single session (Intravisit group). Following informed consent, all subjects were screened for known ocular disease. Participants within the Intervisit group were asked to provide their perceived subjective age (i.e., the age in years they perceive themselves to be) during the initial visit by answering the question: “How old do you feel in years, considering your cognitive and physical performance, your mindset, and your social and professional functionality” [27, 28]. Inter- and Intravisit groups were disjoint; there were no patients present in either group.

### Retinal imaging

All retinal photographs were foveal-centered and conducted in miosis using a Zeiss Visucam Pro NM camera (Carl Zeiss Meditec AG, Jena, Germany). Individuals in the Intervisit group were imaged in two separate visits by a single operator (CS), with the interval between sessions ranging from one to fourteen days. The eyes were repeatedly imaged at each visit until at least one image of sufficient quality (with optimal focus, image field definition, brightness, and artifacts) was available for each eye, as previously described [29]. Participants in the Intravisit group were imaged in a single session, lasting from 4 to 30 min (8 min on average). The order of image acquisition (right or left eye first) was randomly chosen by the examiner and not maintained between the two visits.

### Retinal age predictions and image selection

The DL-based algorithm described previously was used to predict RA from each fundus image. Development, training, and validation of this Algorithm have been described elsewhere [17]. For the evaluation and extraction of image quality variables and vascular morphology features, as well as the initial selection of images based on image quality, we used the open-source deep learning tool AutoMorph [26]. All images were initially evaluated by the quality-control algorithm integrated into AutoMorph to rule in images with sufficient quality without human assessment. Images that did not meet the AutoMorph threshold for image quality were subsequently evaluated by a physician (FT) using objective criteria for image quality previously described by our group and images were ultimately ruled out based on the physicians’ grading of image quality [29]. Thus, images were only included in further analyses if image quality was deemed to be of sufficient quality either by the AutoMorph algorithm, or by the physician if the AutoMorph standards were not met.

### Image quality and test–retest outcomes

Two primary methodologies were utilized to evaluate the impact of image quality on test–retest reliability. Initially, we determined the correlation between the absolute differences of two images from a single eye in all image quality indices provided by AutoMorph (contrast to noise ratio (CNR), colorfulness, contrast, edge-acutance, sharpness, and image-entropy) with test–retest differences of RA predictions. Subsequently, we explored the relationships between the average image quality values of the aforementioned indices and retest reliability. To demonstrate improved retest outcomes when controlling for consistency in image quality, we segregated the images into two subsets based on the median difference in each image quality index available and calculated the resulting mean absolute errors (MAE) in RA prediction for these subsets.

### Diurnal variation in Age-Acceleration of retinal age predictions

To assess the influence of acquisition timing on RA prediction, we correlated hourly discrepancies in image capture times with retest outcomes, disregarding day differences. Additionally, Age-Acceleration (15) (defined as the residuals of the linear regression fit of RA against chronological age) was mapped according to the hour of image acquisition, for the illustration of diurnal variations. For this, Intra- and Intervisit images were each segregated into four distinct groups, based on imaging timepoint and eye laterality. Age-Acceleration was determined from the residuals of individual linear regression models applied to each group [15]. For our primary analysis, we combined Intra- and Intervisit images: Age-Acceleration values were pooled and plotted against image acquisition hour. For data granularity reduction, time points were grouped into morning (07:00–10:59), midday (11:00–13:59), and afternoon (14:00–17:59) intervals. A detailed visualization of this workflow can be found in Supplementary Fig. 1.

### Statistical analysis

All statistical analyses and data visualizations were conducted in R (version 4.30) [30] and Python (version 3.11.2) [31]. Retinal age gap (RAG), defined as the deviation of DLA-predicted RA from chronological age, was calculated as RA minus chronological age. Subjective age gap (SAG) was calculated as the difference between the participant’s subjective age and their respective chronological age. Test–retest differences refer to disparities in DLA predictions of RA from two separate images of a single eye. Linear regressions were performed to assess the correlation between the baseline (from the first available image of a single eye) and follow-up RA prediction (from the second available image), and the intraclass correlation coefficient (ICC) was calculated. Relative standard deviation (RSD) was employed as a measurement of precision and defined as standard deviation (of absolute differences) divided by MAE. For assessing the performance of the algorithm in predicting chronological age (accuracy), predictions of all selected images from one patient (right and left eyes, including the retest sessions) were averaged and correlated with chronological age. To establish a correlation between absolute AutoMorph-derived image quality indices, their discrepancies, and the retest reliability of RA predictions, all image quality metrics provided by AutoMorph were employed [26]. The mean of these indices was computed and correlated with test–retest divergences to provide an average correlation of image quality. For discrepancy analysis, the absolute difference in image quality was calculated and then correlated with retest outcomes. To assess the influence of acquisition timing on RA prediction and diurnal variations in Age-Acceleration, the methodology described above was employed.

Inter-eye consistency was characterized as differences in DLA predictions from images of both eyes from an individual in a single imaging session. Additionally, the retest reliability (defined as deviations in output between two images of a single eye) of all vascular morphology features provided by AutoMorph was correlated with the retest reliability of RA predictions. The respective strength of correlation for all AutoMorph-provided vascular morphology features with retest reliability of RA predictions was depicted in a heatmap. Additionally, for the Intervisit cohort, the correlation between a participant’s SAG and RAG was determined.

Supplementary Table 1 details the number of participants and images included in each analysis, taking into account exclusions based on image quality and inconsistencies in participant data.

For statistical analysis, both descriptive and inferential methods were utilized, guided by the data’s origin and distribution. Descriptive statistics included the calculation of Pearson Correlation Coefficients (PCC) for various purposes: to evaluate the correlation between predicted RA and chronological age, to analyze the relationship between differential and absolute image quality and retest errors, to assess inter-eye correlations, to examine correlations of retest errors in vascular morphology features with RA predictions, and to examine the impact of hourly discrepancies in image acquisition times on test–retest reliability. ICCs were used to assess the correlation of test and retest results of RA predictions from a single eye. Spearman’s Rho was used to correlate SAG with RAG. If multiple correlations were performed, both raw and Benjamini–Hochberg (BH) adjusted *p*-values were reported.

In the realm of inferential statistics, multiple tests were applied as appropriate. Linear regressions were employed to study the relationship between the mean of RA and chronological age and depicted using Bland–Altman plots. The Wilcoxon Rank-Sum Test was used to compare the MAE in the accuracy of RA predictions between the two groups and to analyze the impact of image acquisition order on these predictions, as well as to compare the retest errors across subsets stratified by the median value of each image quality index. For the assessment of circadian variance and the influence of acquisition timing on Age-Acceleration values, both generalized estimating equations (GEE) with an exchangeable structure (for the Intravisit group) and mixed linear effects models (both in the Intervisit group and when pooled) were employed. The goodness-of-fit of this model was compared to a reduced one using likelihood ratio tests. All *p*-values were adjusted using the BH correction for multiple comparisons and BH-adjusted *p*-values were reported.

All statistical tests were two-sided, and a BH-adjusted *p*-value of less than 0.05 was considered to indicate statistical significance.

### Ethics statement

This study was conducted in accordance with the tenets of the Declaration of Helsinki [32] and approved by the Cantonal Ethics Committee with approval number 2022–00947. Informed consent was obtained from all individual participants included in the study.

## Results

### Precision of retinal age predictions

**Fig. 1:**
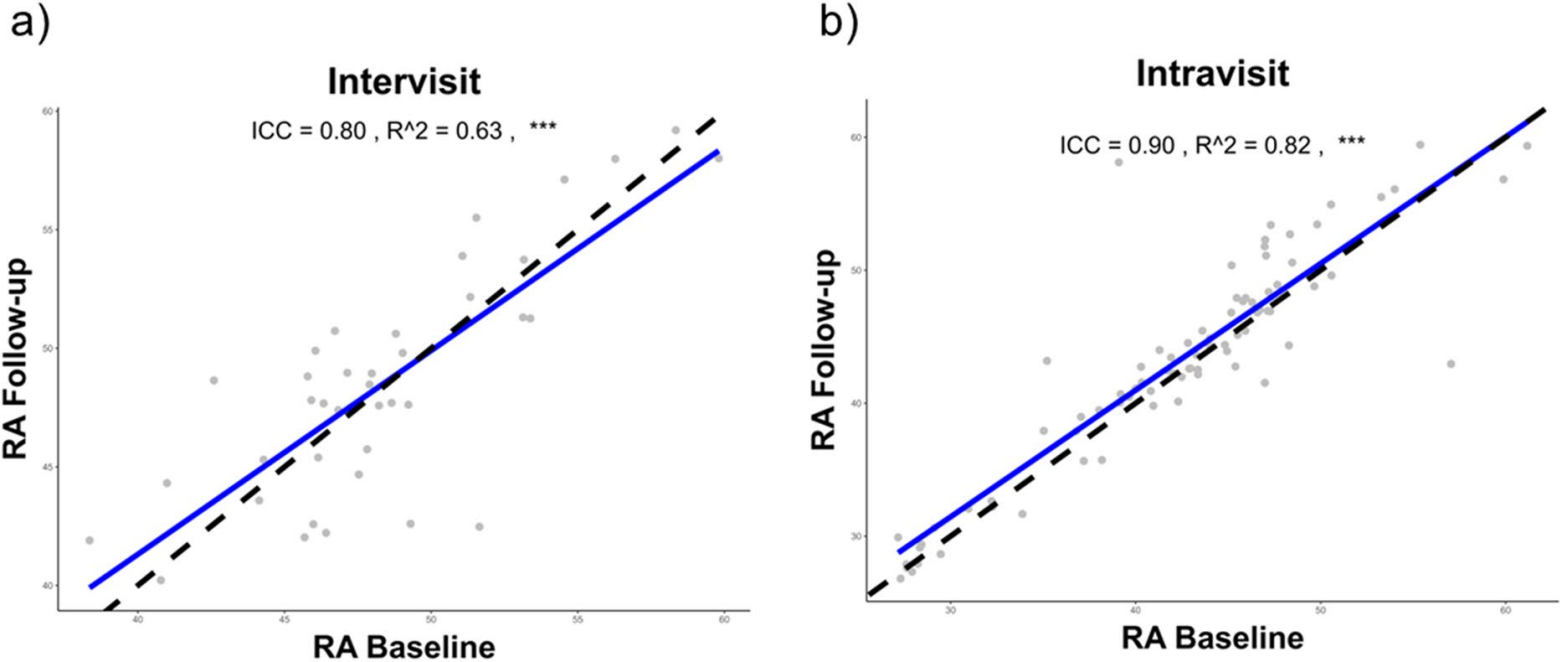
Precision of retinal age predictions. **a** and **b** Scatterplots illustrating predicted RA comparisons between two imaging timepoints of the same eye. **a** Intervisit reproducibility with *n* = 38 image pairs (17 left eyes, 21 right eyes) from 22 patients. **b** Intravisit reproducibility with *n* = 81 image pairs (41 left eyes, 40 right eyes) from 41 patients. The *x*-axis represents the baseline RA prediction, while the *y*-axis shows the RA prediction from the second imaging session. Individual predictions are depicted by grey points. The Line of Unity is denoted by the dashed line, with the blue line indicating the result of the linear regression. RA retinal age, ICC intraclass correlation coefficient; *R*^2, coefficient of determination; ***p-value < 0.001

To determine retest reliability, we computed differences in RA predictions from images taken in a single eye, as illustrated in Fig. 1a and b. These images were either captured twice during one visit (Intravisit) or across two separate visits (Intervisit). After filtering for image quality with AutoMorph and subsequent confirmation by an experienced physician (FT), we excluded two Intravisit and 25 Intervisit images, respectively. Thus, 81 image pairs were available for the Intravisit analysis, and 38 image pairs were suitable for the Intervisit analysis. RA predictions from images of a single eye were highly and significantly correlated in both groups (*p*-value < 0.001, ICC = 0.8–0.9). For the Intravisit group, this correlation was stronger than the Intervisit group, with ICCs of 0.9 and 0.8, and coefficients of determination (*R*^2) of 0.82 and 0.63, respectively. The MAEs for these predictions were 2.13 years for the Intravisit and 2.47 years for the Intervisit group. Regarding prediction variability, the Intravisit group’s RSD was higher at 132.86%, compared to the Intervisit group’s RSD of 79.50%. Notably, large interindividual variability was observed, with some individuals showing tight concordance in predictions, while others displayed substantial discrepancies. In the Intravisit group, absolute differences ranged from 0 to 19 years; in the Intervisit group, they varied from 0.56 to 9.17 years. For a comprehensive overview of all the parameters, see Table 1.

**Table 1:** Key metrics of precision

Metrics	Intervisit	Intravisit
Mean absolute error (MAE, years, %)	2.39, 5.01%	2.1307, 4.68%
SD (absolute age difference, years)	1.90	2.84
RSD (SD/MAE, %)	79.50%	132.86%
Range (age difference, years, min–max)	0.56–9.17	0.01–19.00
ICC, *R*^2, *p*-value	0.80, 0.63, < 0.001	0.90, 0.82, < 0.001

The key metrics of test*–*retest differences: mean absolute error (MAE), absolute standard deviation (SD), relative standard deviation (RSD), range of discrepancies (min, max), intraclass correlation coefficient (ICC), coefficient of determination (*R*^2) and the corresponding *p*-values in the two groups

### Accuracy of retinal age in chronological age predictions

**Fig. 2:**
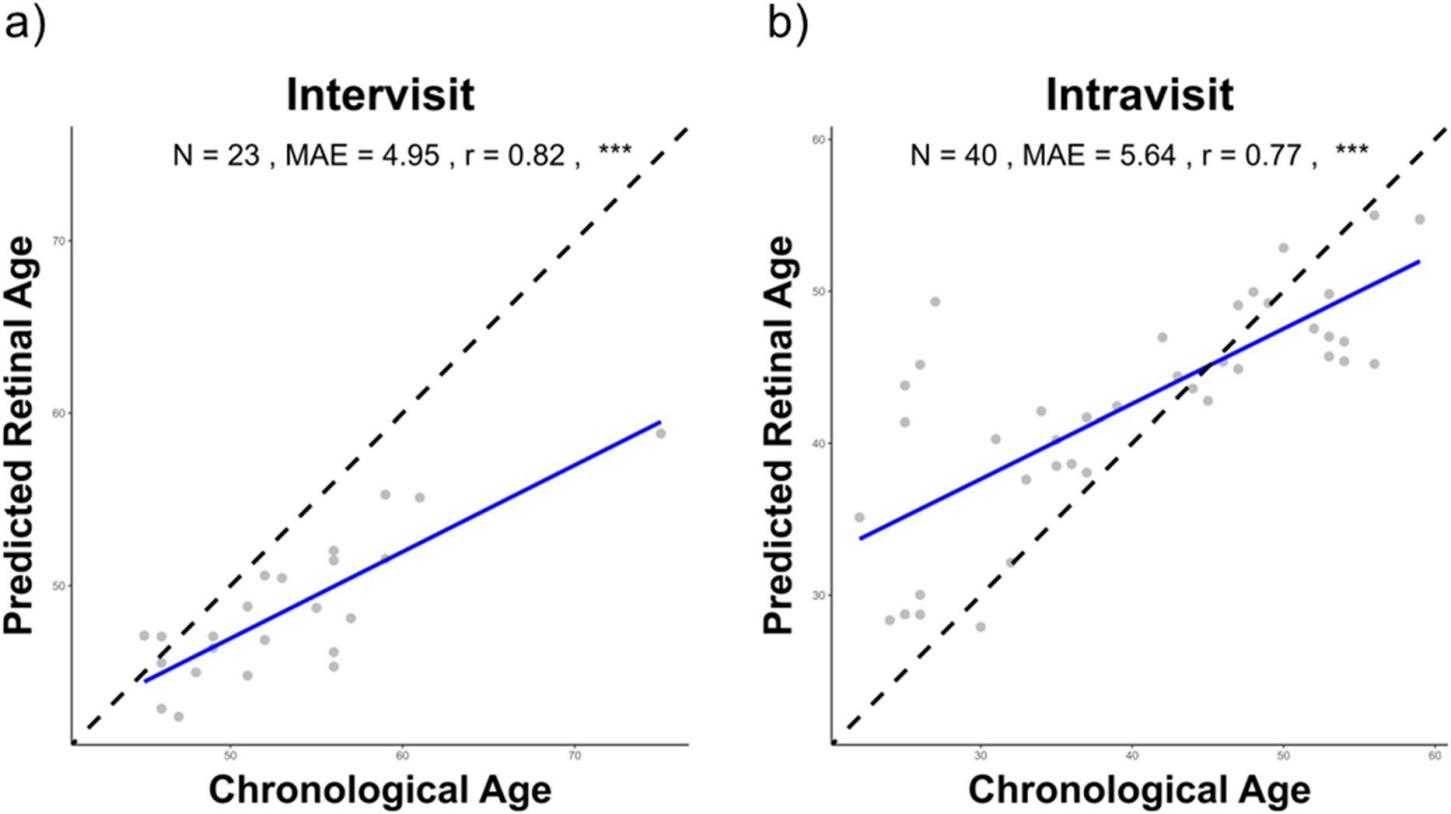
Accuracy of retinal age in chronological age predictions and age acceleration. Scatterplots showing chronological age (*x*-axis) and mean predicted Retinal Age (*y*-axis) for **a** Intervisit (*n* = 23 participants) and **b** Intravisit (*n* = 40 participants) images. Points represent averaged predictions from individual participants, with the solid blue line indicating a linear regression of retinal age against chronological age. The dashed black line depicts the Line of Unity. Key annotations include Pearson’s *r*, mean absolute error (MAE), total count (*N*), and level of significance (***p-value < 0.001)

To evaluate the accuracy of the DLA in predicting chronological age from fundus images of healthy aging individuals, predicted RAs were averaged from all available images for each participant. After excluding images of insufficient quality, and one individual because of inconsistencies in historical data, 40 individuals remained for the Intravisit analysis and 23 for the Intervisit analysis (meaning 3 individuals from the Intervisit cohort lacked any images of sufficient quality). Analyses were conducted separately for each group. Figure 2 displays the correlations of chronological age and RA separately for the two analyses. Both groups had a significant correlation between chronological age and predicted RA, with the Intervisit group showing a stronger correlation (*r* = 0.82, *p* < 0.001) than the Intravisit group (*r* = 0.77, *p* ≤ 0.001). Additionally, the Intravisit MAE of 5.64 years was higher than the Intervisit MAE of 4.95 years, but the differences were not statistically significant (*p* = 0.9718, Wilcoxon rank sum test). A comprehensive overview of all parameters is given in Supplementary Table 2. As shown on the Bland–Altman plot for chronological and RA in Supplementary Fig. 2 (representing all measurements in both cohorts), regression dilution, characterized by age overestimation in younger participants and underestimation in older individuals, was detected. This trend was further substantiated by linear regression analysis, indicating a significant relationship between the mean age of the two measurements and their difference (*r* = 0.69, *p* < 0.001).

### Image quality and retest reliability

**Fig. 3:**
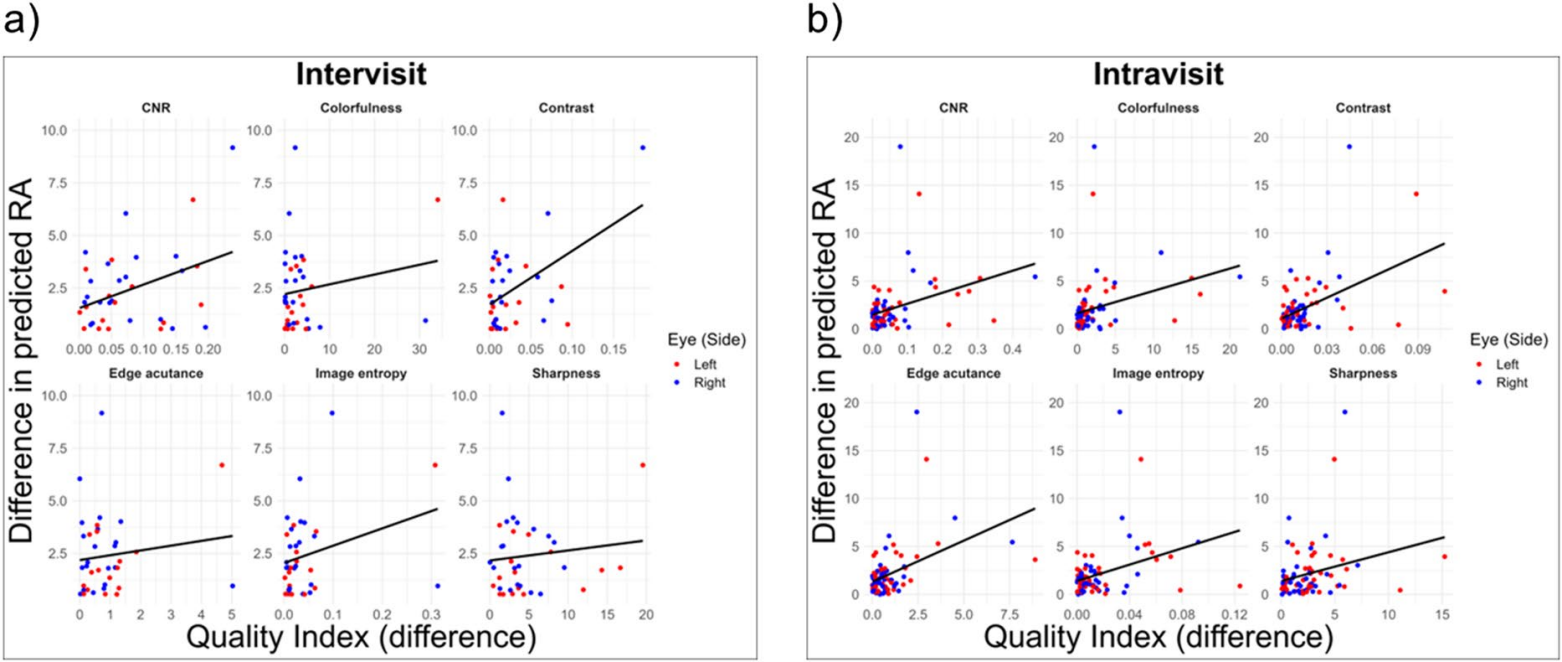
Differential image quality and retest reliability. **a** Intervisit (*n* = 38 image pairs from 22 individuals) and **b** Intravisit (*n* = 81 image pairs from 41 individuals) graphs displaying the correlation between test–retest differences of RA predictions and differential image quality across indices: CNR, colorfulness, contrast, edge-acutance, sharpness, and image entropy. Data points denote individual eyes, with color variations indicating eye laterality (red = left, blue = right). Each graph depicts a particular quality index. Trends are marked by black linear regression lines. CNR contrast-to-noise ratio

In assessing the differential image quality, an increasing difference in image quality was positively correlated with test–retest differences of RA predictions in Intravisit images. This trend was statistically significant and pervasive across all the indices, as shown in Table 2. Notably, the Intervisit images also showed positive correlations between RA prediction differences and differential image quality metrics, but these correlations were weaker in comparison and only significant for divergences in image contrast, as depicted in Fig. 3a. Data from the Intravisit group showed a remarkable clustering in the left lower quadrant of the scatter plots (Fig. 3b). The impact of differential image quality on Intravisit and Intervisit images is further exemplified when subdividing the data based on median absolute differences in image quality indices. For the Intravisit group, a reduction in MAE of RA predictions was observed for the lower 50th percentile across all indices when compared to the overall dataset, proving significant for most metrics of image quality. Reductions in MAE for the lower 50 percentile groups based on differential image quality were: contrast by 31.9% (*p* = 0.142, BH-adjusted), edge acutance by 36.6% (*p* = 0.60, BH-adjusted), sharpness by 29.7% (*p* = 0.04, BH-adjusted), colorfulness by 49.7% (*p* < 0.001, BH-adjusted), CNR by 39.1% (*p* = 0.040, BH-adjusted), and image entropy by 38.5% (*p* = 0.040, BH-adjusted). The most substantial improvement was observed when subsetting by differences in colorfulness, with a decrease in overall MAE of RA predictions from 2.13 to 1.07 years in the lower 50 percentile group, translating to a 49.7% reduction. Similarly, in the Intervisit group, reductions in MAE of RA predictions for the lower 50 percentile were noted across all indices, except for sharpness, though these changes did not reach statistical significance. MAE reductions in the lower 50 percentile were as follows: contrast by 19.6% (*p* = 1, BH-adjusted), edge acutance by 3.1% (*p* = 1, BH-adjusted), colorfulness by 15.1% (*p* = 1, BH-adjusted), CNR by 21.7% (*p* = 1, BH-adjusted), and image entropy by 22% (*p* = 0.978, BH-adjusted). Conversely, when stratifying by the median of differences in image sharpness, the lower 50 percentile group’s MAE showed a 10.0% increase (*p* = 1, BH-adjusted). The most marked improvement in retest outcomes in the Intervisit group was found when stratifying by median image entropy, with a 22% reduction in MAE from 2.38 to 1.854 years. All comparisons were performed using the Wilcoxon rank-sum test, with *p*-values adjusted by the Benjamini–Hochberg method.

**Table 2:** Correlation of differential image quality metrics with test–retest differences

Quality index (differential)	Intervisit		Intravisit	
	PCC	*p*-value (raw, BH-adjusted)	PCC	*p*-value (raw, BH-adjusted)
Colorfulness	0.18	0.29, 0.43	0.31	0.005, 0.006
Contrast	0.5	0.002, 0.009	0.48	< 0.001, < 0.001
Edge acutance	0.13	0.45, 0.5	0.43	< 0.001, < 0.001
Image entropy	0.29	0.0745, 0.145	0.35	0.001, 0.002
Sharpness	0.11	0.5, 0.5	0.26	0.019, 0.02
CNR	0.38	0.017, 0.052	0.35	0.002, 0.003

Pearson correlation coefficients (PCC) and their associated *p*-values (raw and BH-adjusted), comparing differences in image quality of six metrics (CNR, colorfulness, contrast, edge-acutance, sharpness, and image entropy) with the discrepancies in RA predictions. Differential image quality refers to the divergence of a quality metric of the two images used to drive test*–*retest differences in retinal age predictions. The table distinguishes between Intravisit and Intervisit images. A positive PCC implies that as the differences in an image quality metric increase, there is an associated increase in test–retest differences, reflecting reduced precision. On the other hand, a negative PCC would indicate an inverse relationship between the differences in a given quality metric and prediction discrepancies, suggesting that as differential image quality decreases, precision increases

Regarding average image quality, a rise in average image quality for most indices paradoxically was related to higher test–retest discrepancies for both Intravisit and Intervisit images. Interestingly, three indices, including average colorfulness for Intravisit analysis (*r* = − 0.21)), edge acutance, and image entropy in Intervisit analysis (*r* = − 0.12, and r = − 0.05, respectively), diverged from this general trend. A detailed representation of the PCC values and associated *p*-values (both raw and BH-adjusted) across all metrics and analyses is available in Supplementary Table 3.

### Circadian variation of retinal age predictions

**Fig. 4:**
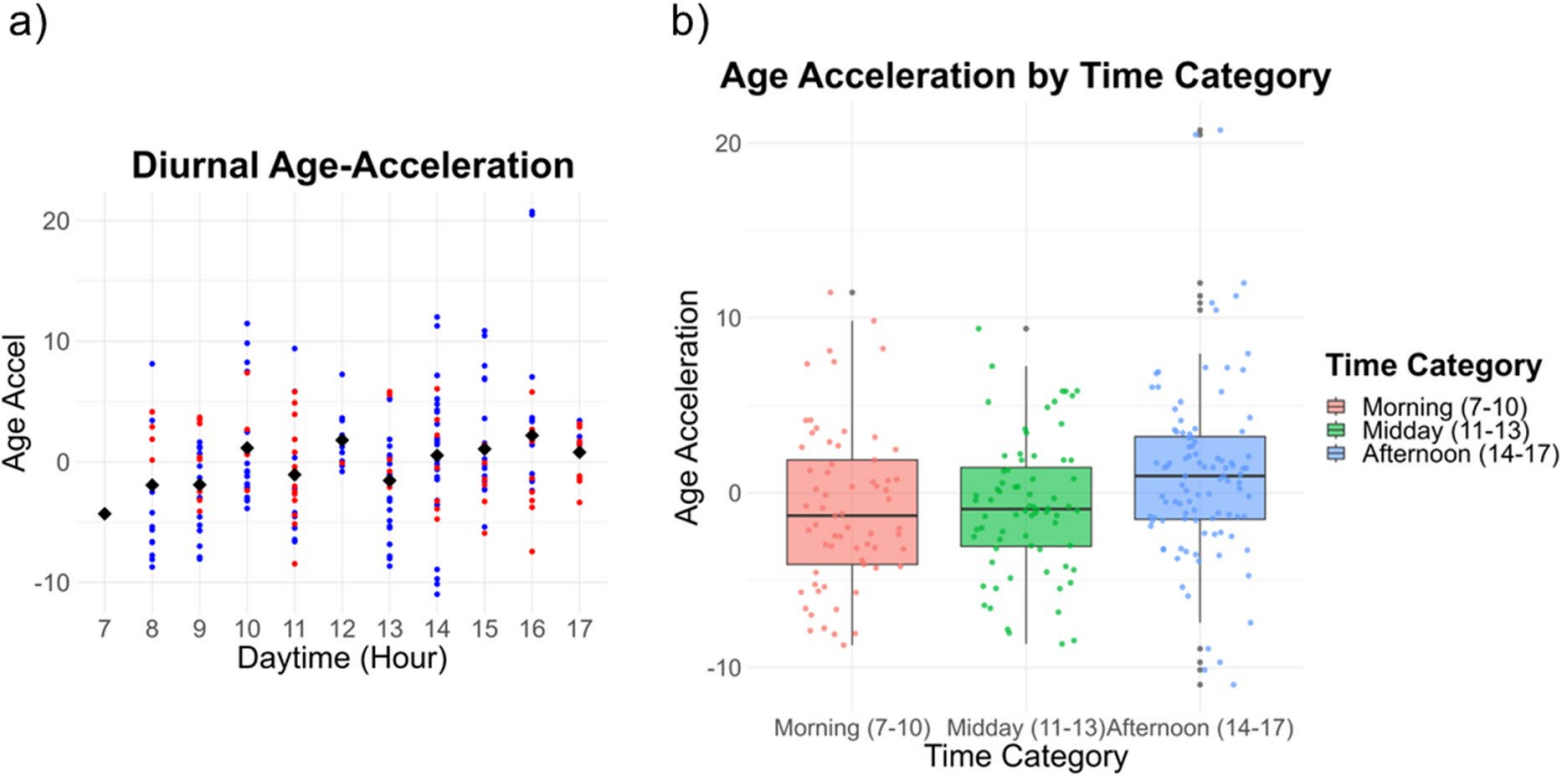
Circadian variation of retinal age predictions. The figure illustrates the circadian variation of Age-Acceleration values based on imaging timepoints, containing Age-Acceleration values from Intravisit (blue, *n* = 158 images from 40 individuals) and Intervisit (red, *n* = 79 images from 23 individuals) images. **a** Age-Acceleration against daytime hours. Each dot represents an image, with mean values highlighted as black squares. **b** visualizes age-acceleration data categorized into three intervals: morning (07:00–10:00), midday (11:00–14:00), and afternoon (14:00–17:59). Displayed are boxplots with overlaid scatterplots. Boxplots indicate the median Age-Acceleration value; the box edges represent the 1st and 3rd quartiles, and the whiskers show the data range within 1.5 times the interquartile range. Data variability is shown by the spread of the box. The analysis encompasses data from a combined total of 237 images from 63 unique individuals

**Fig. 5:**
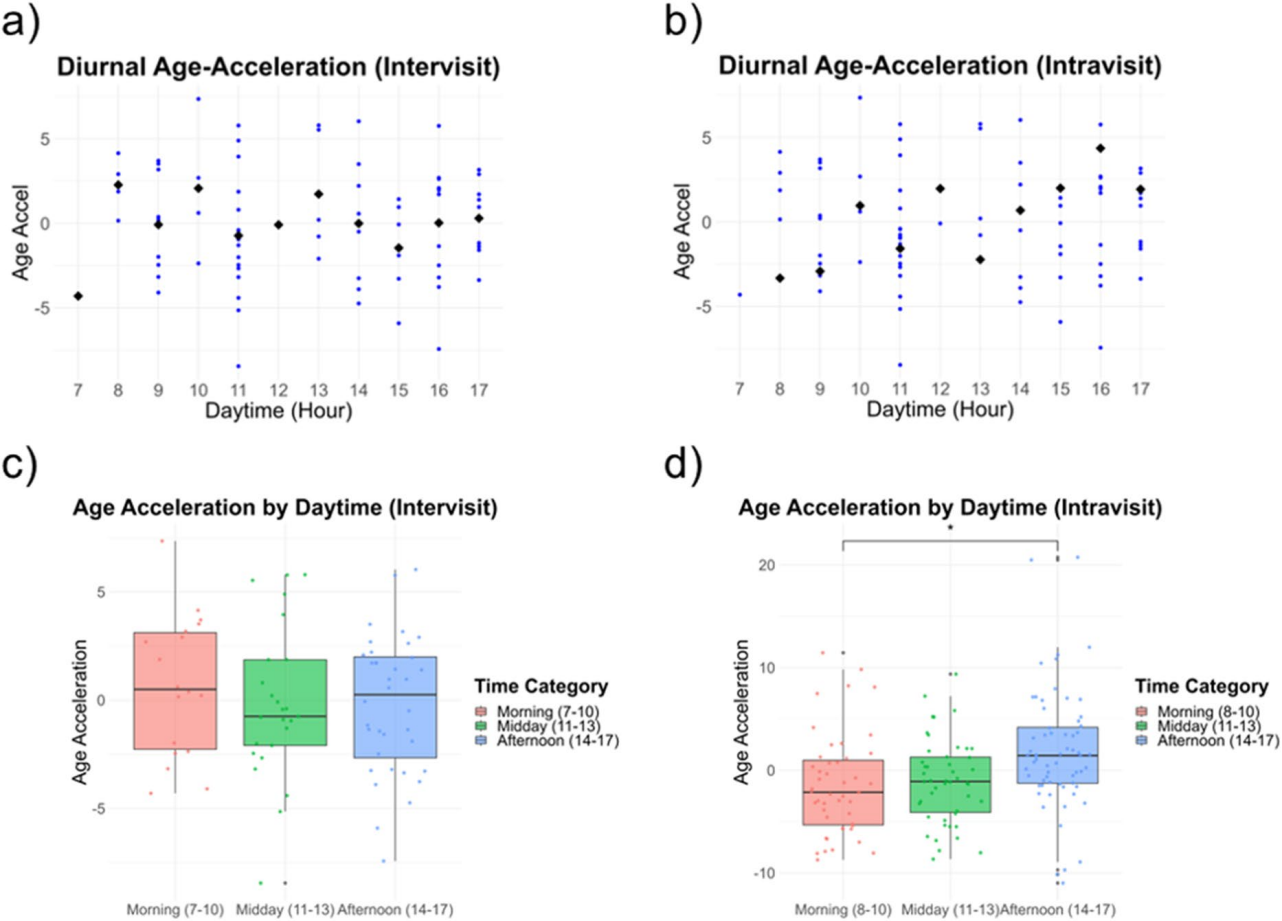
Circadian variation of Age-Acceleration, separate analyses for intra- and intervisit data. Figure displaying the circadian variation in Age-Acceleration, distinguishing between Intervisit (*n* = 79 images from 23 participants) and Intravisit (*n* = 158 images from 40 participants) images. **a** and **b** Scatterplots depicting Age-Acceleration against daytime hours for Intervisit and Intravisit images, respectively. Within these plots, each individual image is symbolized by a blue dot, and the mean Age-Acceleration values are represented by black rhomboids. Further breakdown of Age-Acceleration data is segmented into three daily intervals: morning (07:00–10:00), midday (11:00–14:00), and afternoon (14:00–17:59). **c** and **d** Boxplots illustrating the intervals for Intervisit and Intravisit images, respectively. The central line within each box indicates the median Age-Acceleration, the box edges mark the 1st and 3rd quartiles, and whiskers extend to data within 1.5 times the interquartile range. Scatterplots overlaid on the boxplots emphasize individual data point distributions. *p-value < 0.05

Age-Acceleration values pooled across Intra- and Intervisit groups exhibited a marked diurnal oscillation, as depicted in Fig. 4a. Such oscillatory trends persisted even when the analysis was segregated into Intra- and Intervisit datasets, details of which are elaborated in Fig. 5a and b. In the primary analysis, combining both Intra- and Intervisit data, a subtle but non-significant increase in Age-Acceleration was observed with each advancing hour of the day (estimate = 0.1618 years, *p*-value = 0.6791, mixed linear effects model), as illustrated in Fig. 4a. However, upon categorizing the data into the time intervals of morning, midday and afternoon, no marked significant differences emerged between the groups, as shown in Fig. 4b. This was confirmed by a likelihood ratio test comparing the full model with the “group” predictor to a reduced model with only the intercept (*p*-value = 0.675, mixed linear effects model).

For the Intravisit data, a statistically significant rise in Age-Acceleration values was observed as the day progressed (estimate, 0.6815 years/h; *p*-value = 0.0334; GEE with an exchangeable structure), as depicted in Fig. 5b. Additionally, when segmenting the day into the predefined time intervals, the afternoon group demonstrated a significant elevation in Age-Acceleration compared to the morning (increase, 3.9645 years; *p*-value = 0.0335; GEE, BH-adjusted), as illustrated in Fig. 5d. Conversely, for the Intervisit data, when analyzing the relationship between the acquisition hour and Age-Acceleration, the trend was found to be non-significant (estimate, − 0.0679 years/h; *p*-value = 0.7341; mixed linear effects model), as depicted in Fig. 5a. When evaluating the effect of time intervals (Morning, Midday, and Afternoon) on Age-Acceleration, the mixed linear effects model revealed no significant impact of these grouped timepoints (*p*-value = 0.5137, likelihood ratio test comparing the full model with “group” predictor to a reduced model), as depicted in Fig. 5c.

To evaluate the impact of temporal discrepancies on test–retest reliability, test–retest differences for Intervisit images were correlated with the hour-based differences in image acquisition times across distinct days, while deliberately disregarding the difference in days. No meaningful correlation between these hourly discrepancies and test–retest differences was observed, as indicated by a PCC of 0.02.

### Inter-eye consistency, image order, SAG, and correlation with reliability of retinal vascular features

Correlations between RA predictions from both eyes of a single individual within one visit were notably stronger for Intravisit images (Pearson *r* = 0.81) than for Intervisit images (Pearson *r* = 0.67). Although the MAE for inter-eye consistency was similar at 3.319 years for Intravisit and 3.489 years for Intervisit images, a pronounced difference in variability was evident, with RSD values of 106.5% for Intravisit and 71.5% for Intervisit images, respectively. This variability highlights the broad range in prediction consistency across individuals, with values spanning from 0.133 to 18 years in the Intravisit group and from 0.182 to 8.756 years in the Intervisit group. A comprehensive tabulation of the analytical results across all metrics is provided in Supplementary Table 4.

Additionally, the influence of image acquisition order on test–retest reliability was investigated, classifying order as consistent (same) or varied (different) across imaging sessions. Acquisition order did not significantly affect retest results, with *p*-values of 0.618 and 0.963 for Intravisit and Intervisit, respectively (Wilcoxon rank-sum test).

SAG was not correlated with RAG (Spearman’s Rho = 0.04, *p*-value = 0.84). The mean SAG in the Intervisit group was − 8.72 years (ranging from − 32 to 0 years, with an SD of 8 years), while the mean RAG was − 4.85 years (ranging from − 5 to 5.65 years, with an SD of 4.78 years). Notably, not a single positive SAG was recorded, meaning the perceived subjective age for all participants was equal to or below their chronological age for all participants.

Finally, we assessed the correlation between the retest reliability of RA predictions and the retest reliability of all retinal vascular morphology features extracted using AutoMorph for both Intravisit and Intervisit images. Among the top 10 AutoMorph features that showed the strongest correlation with the retest reliability of retinal age predictions, none were common to both Intravisit and Intervisit analyses. Notably, the absolute PCC did not exceed 0.5 for any feature. The degrees of correlation between the retest reliabilities of retinal age predictions and all AutoMorph features are visualized in Supplementary Fig. 3.

## Discussion

Expanding on the extensively researched concept of RAG as a surrogate biomarker of disease risk and biological age within the framework of the newly coined term, Oculomics [33, 34], this study investigates the various facets of DLA-derived RA predictions. Notably, the DLA employed in this study has recently been shown to surpass virtually most of the other biological age metrics, including epigenetic clocks and neuroimaging, in predicting chronological age in cohorts of healthy individuals and has been extensively validated for its association with clinical outcomes [14, 15, 17]. However, since the algorithm used in previous studies was only validated internally, little is known about the external validity or reproducibility of RA predictions [14, 18–24]. By assessing the algorithm’s precision and accuracy and identifying factors influencing the consistency of RA predictions, our research lays the groundwork for its potential advancement and utility in clinical trials in the near future. To our knowledge, this is the first study investigating the test–retest reliability of any DLA for RA prediction.

The retest reliability analysis indicated significant correlations in RA predictions for both the Intravisit and Intervisit groups. However, the MAE exceeding 2 years in both groups suggests prediction limitations and indicates that detection of subtle changes in biological age over a short time period is not yet achievable. Using retinal age as an endpoint in longitudinal clinical trials will require improved consistency in predictions, allowing the detection of even small variations in biological age over time [15]. Retest reliability and correlation were notably stronger, with a lower MAE for Intravisit images, but surprisingly, Intravisit images also showed higher prediction variability. The lower MAE for the Intravisit analysis suggests that several yet unknown factors, which are held consistent in a single visit but may vary between visits, may influence RA predictions and thus cause higher mean test–retest differences in the Intervisit analysis. Significant interindividual variations in retest outcomes were noted, with some participants showing robust RA predictions and test–retest differences of under 6 months. Identifying factors behind these variations could enhance retest reliability and consistency in RA predictions for subsequent studies.

The DLA’s accuracy in predicting the chronological age from our independent set of fundus images was remarkably consistent with the internal validation outcomes reported for the DLA in its original publication [17]. This congruence underscores the robustness of the algorithm, especially considering that our dataset was independent of the original training and validation sets. However, it is important to note that our findings pertain to a relatively healthy population included in our study. The low prevalence of cardiovascular risk factors among participants may have contributed to the algorithm’s stable performance in our external dataset but may not generalize equally well to broader populations. Despite the presence of up to one cardiovascular risk factor in the Intervisit Group, predictions were more accurate for images stemming from the Intervisit group, compared to Intravisit images. However, differences were not significant, and these discrepancies may likely stem from our small sample size.

Given the imperative for minimal test–retest discrepancies in RA predictions, we identified several factors pertaining to image quality that were notably linked to better retest results. We showed that minimizing the differences in image quality, regardless of whether the image quality is low or high, increases the precision of these predictions. Specifically, by selecting the 50% of image pairs with the least disparities in image colorfulness, we achieved a significant reduction in test–retest discrepancies by 50%. This marks a pivotal advancement towards reliable predictions, thus enabling the detection of genuine changes in biological age over short time periods, a critical requirement for forthcoming clinical trials. The larger correlation between differential image quality and retest discrepancies in Intravisit over Intervisit images again suggests other underlying factors, consistent in Intravisit images but variable in Intervisit images, potentially influencing RA predictions. Though test–retest outcomes from DLAs related to fundus images have been scarcely investigated, our findings indicating the need for consistent data quality, are supported by observations in other fields of machine learning [35].

We also aimed to assess circadian changes in RA measurements, which might represent one of several potential distinctions between the Intravisit and Intervisit groups. While Intravisit images were consistently obtained within a single session not exceeding 30 min, the Intervisit group displayed shifts in the time of day of up to 8 h. Notably, even in the absence of a significant linear correlation between discrepancies in daytime and retest outcomes of RA predictions, we observed diurnal fluctuations in RA predictions, represented as Age-Acceleration values. These observed non-linear oscillations could account for the missing correlation in linear models. Within the Intravisit data, the image acquisition hour served as a significant predictor of Age-Acceleration values, with significantly elevated afternoon predictions compared to morning predictions. This trend was not significant in the smaller Intervisit group. These observations indicated that the circadian timing of image acquisition can influence the degree of age acceleration, suggesting that diurnal variations may lead to deviations from the expected linear relationship between RA and chronological age. The background for our observations is unclear; all subjects were imaged in a standardized fashion, with resting time preceding the imaging sessions; no prandial or continuous blood pressure information was available for the subjects.

Attention maps, which highlight the pixels and areas within CFPs that most strongly contribute to the DLA’s final prediction, suggest that the perivascular regions of fundus images primarily drive DLA-derived estimates of RA [14, 17]. Accordingly, diurnal variations in blood pressure and cerebrovascular features have long been described and may explain the circadian variation in the onset of cardiovascular events such as ischemic strokes [36–44]. Moreover, diurnal variations in retinal and choroidal vessel features and retinal blood flow have also been discovered, but the evidence is still somewhat conflicting [45–48]. Circadian changes in vascular features offer a plausible explanation for observed diurnal variations in RA predictions, underscoring the importance of further research into circadian vascular morphology for improving DLA reproducibility. Given our observations and the potential circadian influence on vascular features, we recommend maintaining consistency in the hour of the day for image acquisition in future clinical studies or commercial applications utilizing DLA algorithms.

Inter-eye consistency differences surpassed 3 years in both Intra- and Intervisit groups. The MAE for Inter-eye consistency exceeded the MAE for test–retest outcomes in both groups, indicating factors beyond just the DLA’s precision. This suggests that besides systemic biological factors influencing RA, local eye-specific factors also play a role in RA predictions. However, the examined factors, including image acquisition order and retest outcomes of AutoMorph extracted features, showed weak correlations with RA prediction retest results. Hence, we see no immediate necessity to standardize these factors for the DLA’s forthcoming application.

Our study has certain limitations. While we acknowledge the study’s limited sample size and image count, this is in line with most reproducibility studies, and we thus believe it sufficiently estimates the precision of RA predictions [49–57]. For a comprehensive external validation of accuracy, larger image datasets will be essential, and the observed diurnal variations and image quality effects in this study still merit deeper exploration. We aimed to maintain a standardized procedure during image acquisition in order to minimize variation, and thus believe this did not influence our results. As we were primarily aiming to assess the reproducibility of RAG measurements, we did not collect further data on the patients, such as prandial status, blood pressure, or subjective stress levels. Nevertheless, we are confident that our research offers a valuable estimation of precision, offering critical insights for the design of future clinical trials employing this DLA.

In conclusion, we showed that RAG predictions via the DLA achieved moderate precision, and maintaining consistent image quality could potentially improve the reliability of RA predictions. Additionally, we uncovered possible diurnal variations in RA predictions. These insights constitute an essential step towards further improvement of RA predictions within the context of Oculomics and mark an essential step towards the application of this DLA in clinical trials.

## Supplementary Information

Below is the link to the electronic supplementary material.

Supplementary file1 (DOCX 914 KB)
