# Supplementary material for: Evaluating the reproducibility of a deep learning algorithm for the prediction of retinal age
Source: GeroScience. 2024 Nov 26;47(2):2541–54. doi: 10.1007/s11357-024-01445-0 (PMC11979088; doi:10.1007/s11357-024-01445-0)
Supplement: Supplementary file 1 — Supplementary file1 (DOCX 914 KB) [file 11357_2024_1445_MOESM1_ESM.docx]

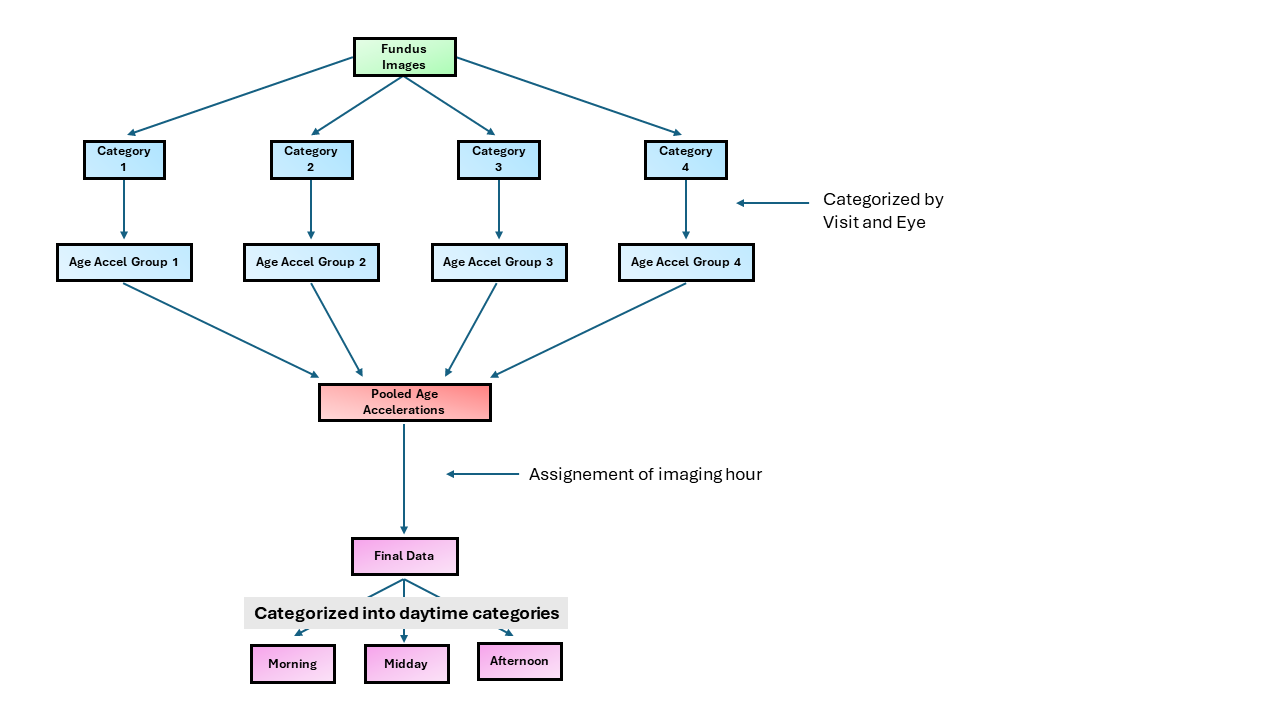


**Supplementary Fig. 1. Workflow for Age-Acceleration.** *Analysis was conducted separately for Intravisit, Intervisit, and also combined images. Noting poor test-retest reliability and inter-eye consistency, fundus images from the Intravisit and Intervisit groups were each stratified into four distinct subsets based on eye laterality and imaging timepoint. Baseline Image refers to the first image acquired from a single eye, either within a single visit (Intravisit) or in the first visit (Intervisit). Residuals from the respective linear regression model, separately applied to each subcategory, defined Age-Acceleration values. Data from these subsets were then aggregated. Age-Acceleration was mapped according to the hour of image acquisition. For granularity reduction, values were grouped into three specific time intervals: Morning (07:00 - 10:00), Midday (11:00 - 14:00), Afternoon (14:00 - 17:00).*

| **Corresponding Experiment** | **Intervisit** | | **Intravisit** | |
| --- | --- | --- | --- | --- |
|  | **Number of Individuals (n)** | **Number of Images (Total, Pairs)**  (raw, BH-adjusted) | **Number of Individuals (n)** | **Number of Images (Total, Pairs)**  (raw, BH-adjusted) |
| **Total** | **26** | **104** | **41** | **164** |
| **After Filtering for Image Quality** | **23** | **79 Images, NA** | **41** | **162 Images, NA** |
| 3.1 Precision of Retinal Age Prediction | 22 | 76 Images, 38 Pairs | 41 | 162 Images, 81 Pairs |
| 3.2 Accuracy of Retinal Age in Chronological Age Predictions | 23 | 79 Images, NA | 40 | 158 Images, NA |
| 3.3 Image Quality and Retest-Reliability | 22 | 76 Images, 38 Pairs | 41 | 162 Images, 81 Pairs |
| 3.4 Circadian Variation of Retinal Age Predictions | 23 | 79 Images, NA | 40 | 158 Images, NA |
| 3.5 Inter-Eye Consistency | 17 | 66 Images, 33 Pairs | 40 | 160 Images, 80 Pairs |
| 3.5 Image Order | 22 | 76 Images, 38 Pairs | 41 | 162 Images, 81 Pairs |
| 3.5 SAG | 23 | 79 Images, NA | 40 | 158 Images, NA |
| 3.5 Correlation with Reliability of Retinal Vascular Features | 22 | 76 Images, 38 Pairs | 41 | 162 Images, 81 Pairs |

**Supplementary Table 1. Distribution of Participants and Image Counts Across Experiments.** Table summarizing the total number of participants and images included in each experiment across both the Intervisit and Intravisit cohorts. For each experiment, the table details the number of individuals and corresponding images (total images and image pairs where applicable) after filtering for image quality. The fluctuation in image counts is attributable to the requirement for paired images in certain experiments and the exclusion of one individual from the Intravisit cohort in all experiments involving chronological age.

NA: Not Applicable.

*
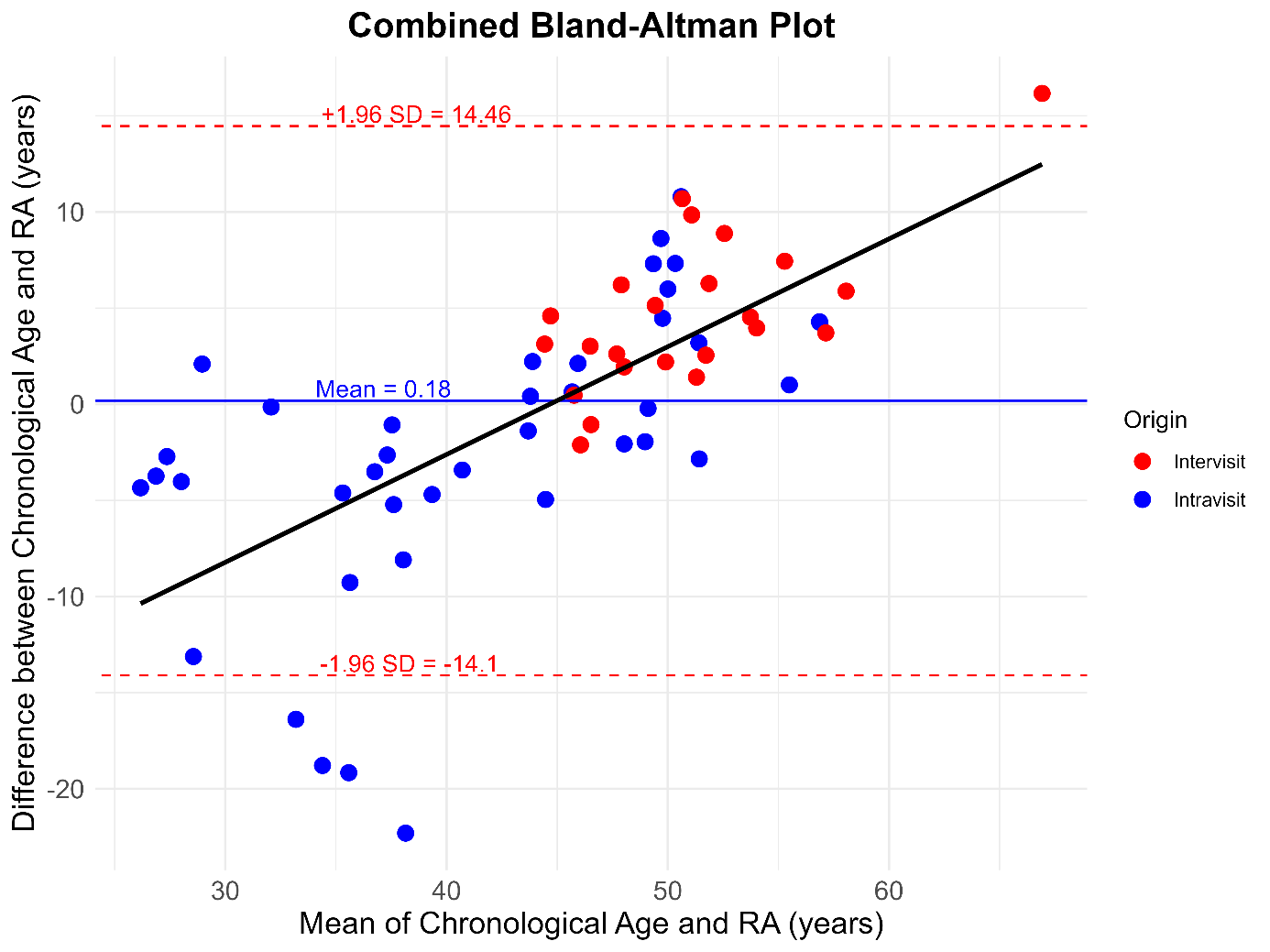
*

**Supplementary Fig. 2. Combined Bland-Altman Plot Illustrating the Agreement Between Chronological Age and Average Predicted Retinal Age (RA).** *The Intravisit analysis (n=40) is represented in blue, and the Intervisit analysis (n=23) in red. The blue line represents the mean difference, with the 95% limits of agreement (±1.96 SD) indicated by dashed red lines. The linear regression line is depicted by the solid black line. Notably, for every year increase in the mean age, the difference between the chronological and RA increases by approximately 0.56 years (r= 0.69, p<0.001, linear regression analysis), representing regression dilution, characterized by age overestimation in younger participants and underestimation in older ones.*

| **Metrics** | **Intervisit** | **Intravisit** |
| --- | --- | --- |
| **Chronological age (mean, range, years)** | 53.3 (45-75) | 40.4 (22-59) |
| **Retinal Age prediction (mean, range, years)** | 48.6 (42.4- 58.9) | 42.8 (27.9-55) |
| **MAE (years, (%))** | 4.95 (8.82%) | 5.64 (17.34%) |
| **SD (absolute, years)** | 3.7 | 5.4 |
| **RSD(%)** | 75% | 96% |
| **Range (error , min-max, years)** | 0.47 – 16.2 | 0.14 - 22 |
| **Pearson r, p-value** | 0.82, <0.001 | 0.77, <0.001 |

**Supplementary Table 2. Accuracy of Retinal Age in Chronological Age Prediction.** *Table presenting key metrics of accuracy of RA prediction separately for the Intervisit and Intravisit groups. MAE: mean absolute, SD: standard deviation of absolute errors, RSD: Relative standard deviation, Pearson r: Pearson Correlation Coefficients and their associated p-values.*

*CNR: Contrast-to-noise ratio.*

| **Quality Index (average)** | **Intervisit** | | **Intravisit** | |
| --- | --- | --- | --- | --- |
|  | **PCC** | **p-value**  (raw, BH-adjusted) | **PCC** | **p-value**  (raw, BH-adjusted) |
| Colorfulness | 0.12 | 0.470, 0.705 | -0.21 | 0.057, 0.171 |
| Contrast | 0.30 | 0.068, 0.205 | 0.16 | 0.170, 0.340 |
| Edge acutance | -0.12 | 0.468, 0.705 | 0.32 | 0.004, 0.0024 |
| Image entropy | -0.05 | 0.785, 0.830 | 0.11 | 0.330, 0.369 |
| Sharpness | 0.31 | 0.056, 0.205 | 0.07 | 0.520, 0.52 |
| CNR | 0.04 | 0.830, 0.830 | 0.11 | 0.320, 0.396 |

**Supplementary Table 3. Correlation of Average Image Quality Metrics with Test-Retest Differences.** *Pearson Correlation Coefficients (PCC) and their associated p-values (raw and BH-adjusted), comparing average image quality of six metrics (CNR, Colorfulness, Contrast, Edge-Acutance, Sharpness, and Image-Entropy) with the discrepancies in RA predictions. Average image quality refers to the mean of a quality metric of the two images used to drive test-retest differences in Retinal Age predictions. The table distinguishes between Intravisit and Intervisit images. A positive PCC implies that as the average image quality metric increases, there is an associated increase in test-retest differences, reflecting reduced precision. On the other hand, a negative PCC indicates an inverse relationship between the given quality metric and prediction discrepancies, suggesting that as average image quality decreases, precision increases. CNR: Contrast to noise ratio.*

| **Metrices** | **Intravisit** | **Intervisit** |
| --- | --- | --- |
| MAE (inter-eye difference) | 3.3198 | 3.489 |
| SD (absolute age difference) | 3.53477 | 2.495 |
| RSD (SD/MAE, %) | 106.5% | 71.5% |
| Range (error, years, min-max) | 0.133-18 | 0.182-8.756 |
| Pearson r, p-value | 0.81, <0.001 | 0.67, <0.001 |

**Supplementary Table 4. Inter-Eye Consistency Metrics.** *Table showcasing the inter-eye consistency of RA predictions between the two eyes of an individual captured during a single imaging session. For the Intravisit category, data were derived from 80 image pairs, each representing both eyes at a given time point. The Intervisit category was based on 33 image pairs, split into 16 image pairs for Baseline and 17 for Follow-up. Metrics include the Mean Absolute Error (MAE) inter-eye difference, which represents the average deviation in age predictions between the eyes; the Standard Deviation (SD) of the absolute age difference; the Relative Standard Deviation (RSD), computed as MAE over SD; as well as the minimum and maximum observed age prediction differences. Additionally, the table provides the Pearson correlation coefficient and its associated p-value.*

**
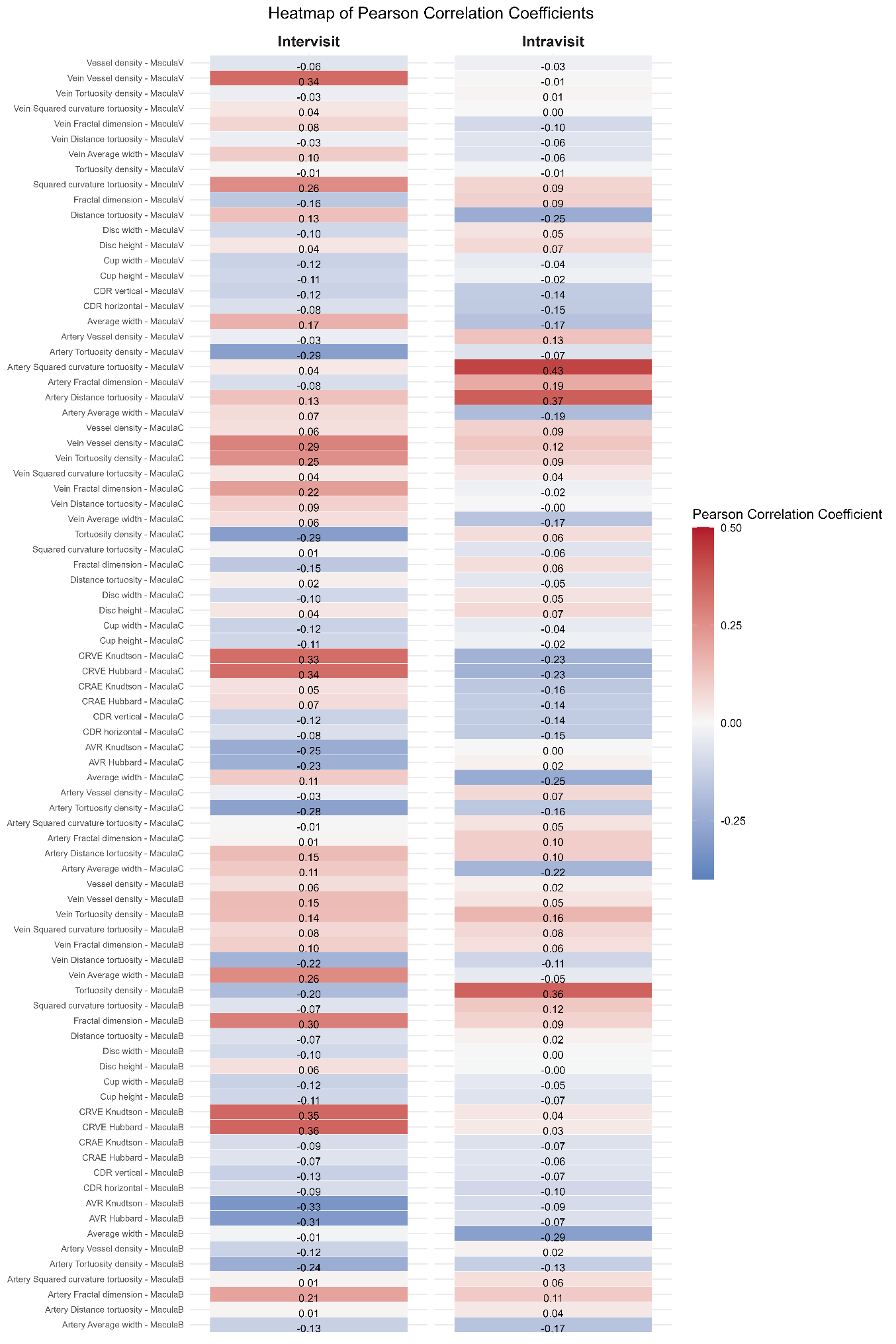
**

**Supplementary Fig. 3. Pearson Correlation Coefficients for Test-Retest Differences of Vascular Morphology Features and Retinal Age Predictions.** *Heatmap visualizing the PCCs derived from analyzing test-retest differences in Retinal Age predictions alongside vascular morphology features. To obtain these correlations, we first computed the test-retest differences for both RA predictions and each vascular feature individually. Subsequently, PCCs were calculated between the test-retest variations of RA predictions and every individual vascular morphology feature. The color gradient in the heatmap indicates the strength and direction of the correlation, with blue representing negative correlations and red signifying positive correlations. The annotations within each cell provide the exact PCC value. The horizontal axis differentiates between Intervisit and Intravisit analysis.*
